# AI-Enabled regional tele-ECG cloud platform and improving access to cardiovascular diagnosis: real-world evidence from southern China

**DOI:** 10.3389/fcvm.2026.1869716

**Published:** 2026-07-01

**Authors:** Jia Xu, Min Pan, Lin Chen, Juan Fu, Rui Shi, Jun Xie

**Affiliations:** 1 Department of IT & Data Management of West China Hospital, Sichuan University, Chengdu, China; 2 Laboratory of Medical Artificial Intelligence, West China Hospital, Sichuan University, Chengdu, China; 3 Department of Cardiovascular Medicine, Sanya People's Hospital, Sanya, China; 4 West China (Sanya) Hospital, Sichuan University, Sanya, China; 5 Department of Cardiocerebral Function, Sanya People's Hospital, Sanya, China; 6 Information Center, Sanya People's Hospital, Sanya, China

**Keywords:** artificial intelligence, budget impact analysis, digital health infrastructure, healthcare accessibility, real-world evidence, tele-cardiology, workflow reengineering

## Abstract

**Background:**

Significant disparities in cardiovascular disease outcomes persist between urban centers and resource-constrained primary healthcare (PHC) settings due to geographic barriers and uneven access to specialist expertise. Digital health networks offer a potential strategy to improve diagnostic accessibility. This study evaluated the real-world implementation of an AI-enabled regional tele-ECG cloud platform within an integrated urban medical group.

**Methods:**

A longitudinal real-world evaluation was conducted using 1,998 tele-ECG transmissions, including 53 confirmed myocardial infarction (MI) cases at the PHC level within a network of 1,166 MI patients. The platform integrated AI-assisted ECG interpretation with centralized specialist review through a cloud-based B/S architecture. Analyses included inter-tier comparisons, subgroup analyses by geographic location and age (≥65 years), *post-hoc* power assessment, and a Budget Impact Analysis (BIA) with sensitivity analysis.

**Results:**

Platform implementation was associated with reduced delays in emergency cardiovascular care. Among patients presenting with chest pain at PHC institutions, median clinical decision-making time decreased from 10 to 3 min (70.0% reduction), while report turnaround time (TAT) was 3.79 ± 1.81 min. No significant differences were observed between PHC institutions and the tertiary hospital in TAT (*P* = 0.4384) or report review times (*P* = 0.8102). Diagnostic accuracy at the PHC level increased from 82.30% to 98.11%. PHC institutions achieved a survival-to-discharge rate of 75.00% among acute MI cases. The BIA showed an average patient saving of 26 CNY per encounter and an annual net social benefit of 154,182.50 CNY for the regional network.

**Conclusions:**

The AI-enabled regional collaborative model was associated with improved access to cardiovascular diagnosis across geographically diverse settings. Despite limited statistical power in the PHC subgroup (mean power: 32.4%) and potential confounding from seasonal population migration, the platform may provide a scalable approach for strengthening cardiovascular diagnostic capacity in resource-constrained regions.

## 1 Introduction

Cardiovascular diseases (CVDs) remain the leading cause of death worldwide. According to the World Health Organization (WHO), approximately 19.8 million people died from CVDs in 2022, accounting for 32% of all global deaths [cf (1).]. The burden is particularly pronounced in low- and middle-income countries, where timely identification and intervention are essential for reducing morbidity and mortality. In China, the number of individuals living with CVDs has exceeded 330 million, with an increasing trend toward younger age groups [cf (2).].At the same time, inequities in healthcare resource distribution continue to contribute to disparities in cardiovascular outcomes. Since 2009, CVD mortality rates in rural China have consistently exceeded those in urban areas. This persistent urban–rural disparity highlights longstanding differences in access to timely cardiovascular diagnosis and treatment, reflecting challenges faced by primary healthcare institutions in managing acute cardiovascular events. Improving access to high-quality cardiovascular diagnostic services across geographically diverse settings has therefore become an important public health priority for promoting health equity within regional healthcare networks [cf (2, 3).].

From the perspective of the social determinants of health (SDOH), insufficient geographic accessibility and a shortage of specialized human resources are the core bottlenecks restricting primary cardiovascular prevention and control capabilities [cf (4).]. Primary healthcare (PHC) institutions generally lack specialized cardiovascular support, resulting in limited capacity for the identification and management of acute events. Time-dependent diseases, exemplified by ST-segment elevation myocardial infarction (STEMI), rely heavily on rapid diagnosis. However, PHC institutions often face a “diagnostic dilemma”, where the misdiagnosis rate of electrocardiograms (ECGs) by community physicians reaches as high as 10.5%. Coupled with the “information silo” effect caused by inadequate connectivity between pre-hospital and in-hospital systems, many patients miss the “golden window” for treatment[cf (3, 5–9).].

The WHO Global Strategy on Digital Health emphasizes that digital technology serves as a powerful lever for breaking down geographic barriers and achieving health equity [cf (4).]. In recent years, digital health technologies—centered on 5G, cloud computing, and artificial intelligence (AI)—have provided a new pathway for reconstructing regional cardiovascular prevention networks [cf (7, 10–13).]. Practices in Minas Gerais, Brazil, have demonstrated that collaborative networks can prevent 81% of unnecessary referrals [cf (14, 15).]; similarly, a STEMI network established in Egypt significantly shortened door-to-balloon (D2B) time and reduced in-hospital mortality [cf (16, 17).]. While China has promoted the construction of “ECG networks, ” existing research has primarily focused on technical feasibility. There is a lack of in-depth evaluation regarding how intelligent models reshape regional health equity and provide systemic empowerment [cf (14, 18).].

As a typical coastal tourist city, Sanya faces the dual pressure of a local aging population and a mobile population of 200, 000 seasonal sojourners, making it an ideal sample for assessing the social outcomes of smart healthcare. This study uses the Sanya regional tele-ECG cloud platform as a case study to explore the impact of an intervention model based on B/S architecture and an AI engine on public health outcomes. The objective is to determine whether a digital platform can enhance primary diagnostic quality, reduce rescue delays, improve clinical outcomes, and provide quantifiable health economic benefits. The findings aim to provide empirical support for sustainable public health governance in resource-limited regions worldwide.

## 2 Methods

### 2.1 Study design and data sources

**Figure 1:**
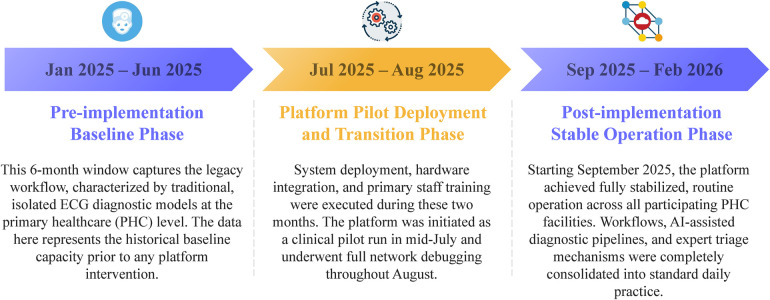
Project implementation timeline diagram.

This study adopts a longitudinal real-world evaluation to assess the utilization trajectories of the regional tele-ECG platform. To systematically control for Sanya's unique macro-demographic seasonal fluctuations (i. e., the winter sojourner influx), the tracking window was expanded to 14 consecutive months from January 2025 to February 2026.The timeline is partitioned into three sequential phases (Figure 1): (1) the pre-implementation baseline phase (January to June 2025, reflecting the traditional isolated diagnostic model); (2) the platform pilot deployment and transition phase (July to August 2025); and (3) the post-implementation stable operation phase (September 2025 to February 2026).

Definition of Myocardial Infarction (MI):The case definitions utilized in this study strictly adhered to the clinical criteria derived from the Fourth Universal Definition of Myocardial Infarction (2018), and a confirmed diagnosis required the independent professional judgment of attending cardiologists grounded on the following standardized multi-dimensional criteria, with entirely blinded to the AI output: a mandatory biomarker elevation requirement of a rise and/or fall of cardiac troponin (cTnI or cTnT) with at least one value exceeding the 99th percentile upper reference limit (URL), which must be combined with concomitant clinical evidence including symptoms of acute myocardial ischemia such as acute ischemic chest pain, new ischemic ECG changes involving dynamic ST-segment or T-wave evolutions or the development of pathological Q waves, imaging evidence of new loss of viable myocardium or new regional wall motion abnormality, or angiographic confirmation of an intracoronary thrombus via coronary angiography (CAG) where applicable.

Data Sources: A dual-track data collection strategy, combining “automated system extraction” and “retrospective clinical surveys,” was applied across 12 public medical institutions (including 11 primary healthcare centers) within the platform network. Core quality control indicators, such as ECG report turnaround time (TAT), post-implementation diagnostic accuracy, and AI-assisted audit error rates, were directly extracted from the platform to ensure objectivity. Non-diagnostic indicators within the myocardial infarction (MI) care pathway—including door-to-needle (D2N) time, clinical decision-making time, and prognostic outcomes—were collected through retrospective manual surveys involving structured interviews with 11 primary care physicians. This approach was necessitated by data confidentiality restrictions at primary hospitals and the current lack of full automated integration between the platform and various primary hospital information systems (HIS). To mitigate potential recall bias associated with manual surveys, a consistency verification strategy was implemented: two researchers independently entered the survey data, and random samples were cross-checked against original records to ensure data quality.

Inclusion Criteria: (1) Patients who completed an ECG examination within the platform-covered institutions and were clinically diagnosed with myocardial infarction (MI); (2) patients with complete clinical diagnostic records and follow-up data.

Exclusion Criteria: (1) Patients transferred from external hospitals who had already completed a preliminary diagnosis; (2) patients with missing key emergency time points (e. g., ECG acquisition time); (3) patients who explicitly refused the use of their data for research purposes.

Ethical Statement: This study strictly adhered to the Declaration of Helsinki. All data were de-identified and anonymized prior to extraction. The study received approval from the Ethics Committee of Sanya People's Hospital (the lead institution of the platform) (Approval No.: 2026 (28)).

### 2.2 System architecture: A Low-cost adaptation scheme for resource-constrained regions

**Figure 2:**
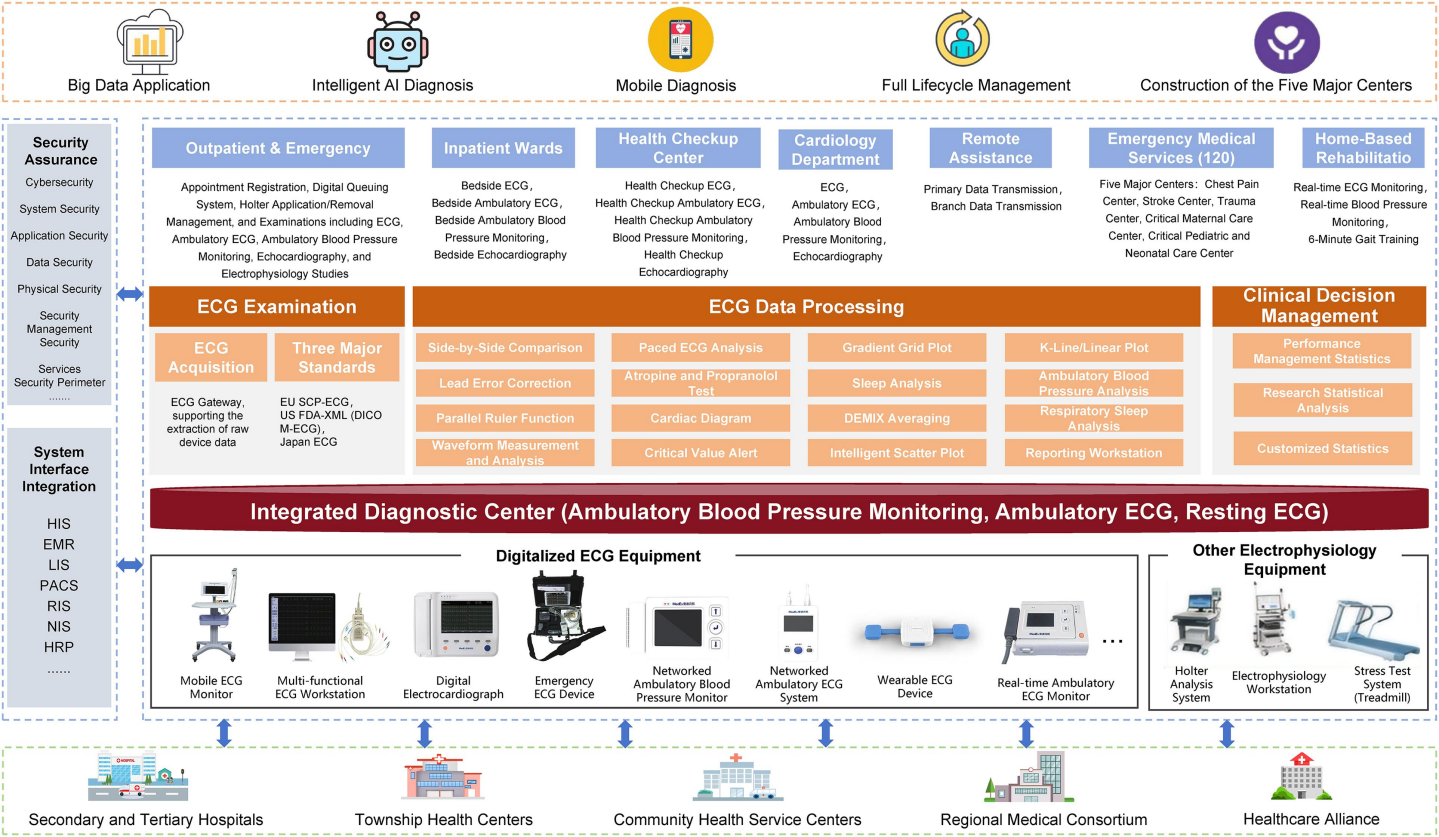
System architecture.

To address the challenges of limited operational funding and weak maintenance capabilities in primary healthcare (PHC) institutions, this study developed a cloud architecture based on the Browser/Server (B/S) model [cf (19–23).]. Through “lightweight” deployment, the platform maximizes the utility of existing legacy equipment in primary hospitals, allowing patients to access expert diagnostics locally. This approach reduces medical expenditures, strengthens primary-level first-contact care, and effectively lowers both initial construction and long-term maintenance costs. The logical architecture of the platform is divided into four core layers (Figure 2):

Perception and Access Layer: This layer integrates a standard conversion engine supporting international protocols such as SCP-ECG, DICOM, and FDA-XML. It achieves compatibility with both legacy devices and new wearable sensors, thereby lowering the financial threshold for hardware upgrades.

Secure Transmission Layer: Utilizing 5G medical private networks to construct encrypted tunnels, this layer ensures the secure transmission of public health big data across complex geographical environments.

Cloud Processing and Support Layer: Deployed at the regional center, this layer integrates the AI-assisted diagnostic engine. It is responsible for the centralized storage, standardized management, and intelligent interpretation of massive ECG data volumes.

Application and Presentation Layer: Supporting multi-terminal access without the need for software installation, this layer allows users to log in directly via a web browser. This achieves the “spatiotemporal decoupling” of diagnostic workflows and provides mobile professional support for primary care physicians.

### 2.3 Web-Based tele-ECG infrastructure, AI-driven triage, and quality control engine

To mitigate geographic disparities in diagnostic timeliness and address the “black-box” limitations, the platform implements a regional collaborative workflow powered by a Browser/Server (B/S) architecture and an integrated technical-clinical triage engine.

The underlying twelve-lead ECG artificial intelligence core is a commercialized solution developed by a medical technology enterprise, operating as a preliminary filtering and real-time alert gatekeeper within the network. Its granular architectural parameters and training boundaries are specified as follows:

Adaptive Preprocessing: To address the severe artifact distortions common in legacy or low-sampling-rate acquisition equipment deployed at grassroots primary healthcare (PHC) clinics, raw ECG signals undergo adaptive preprocessing via a deep autoencoder-based denoising module. This module performs real-time baseline wander correction and digital band-pass filtering to eliminate power-line interference (50/60 Hz), electromyographic (EMG) noise, and high-frequency motion artifacts (20–23).

Hybrid Deep Topology: The denoised multi-channel time series are processed by a serial hybrid deep neural network consisting of a 7-layer 1D Convolutional Neural Network (1D-CNN) feature encoder to capture localized wave morphologies (e. g., QRS notches or subtle ST-segment offsets), coupled with a 12-layer Transformer Encoder stack utilizing multi-head self-attention mechanisms to model long-range temporal dependencies across distinct cardiac cycles. The network terminates in a fully connected projection head with a Sigmoid activation to output independent classification probabilities [P∈(0, 1)].

Pre-training & Fine-tuning Framework: The model's foundational robustness was established via a Self-Supervised Contrastive Learning (SimCLR) framework on a multi-vendor heterogeneous dataset of 1.5 million 12-lead ECG records (aggregating the UK Biobank, MIMIC-IV-ECG, CODE-15, PTB-XL, and PhysioNet 2021 Challenge). Stochastic data augmentations—including mild amplitude scaling, random temporal cropping, and sequence masking—were optimized using an InfoNCE loss function. Supervised fine-tuning was subsequently performed on a high-quality, private, expert-annotated database of over 100, 000 unique records across 44 diagnostic abnormality categories, using a strict 7:1:2 patient-level split to completely isolate intra-patient data leakage between the training and testing matrices. Due to commercial proprietary and Intellectual Property (IP) constraints, the internal layer weights remain restricted.

Decision Thresholds & Technical Performance Bounds: Classification thresholds were determined automatically by maximizing the Youden Index on the validation set and subsequently frozen; no *post-hoc* probability calibration was applied. Under closed technical validation on the developer's test set, the underlying engine demonstrated high discriminative capacities [e. g., an Area Under the Receiver Operating Characteristic curve [AUC] of 0.996 for Atrial Fibrillation and 0.974 for Premature Ventricular Contractions [PVCs]].

**Figure 3:**
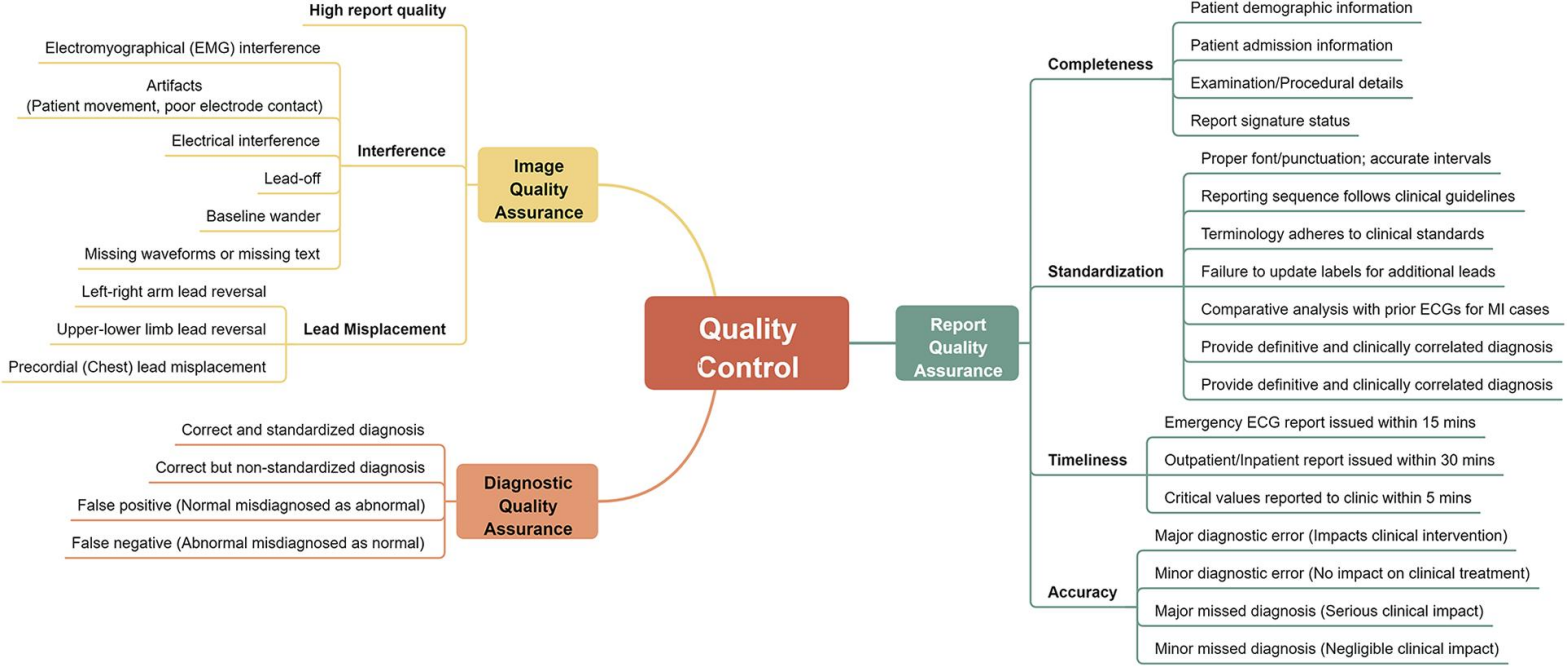
Quality control metrics.

Real-Time Quality Control Engine: To mitigate diagnostic errors caused by improper operational maneuvers or paramedic fatigue at the primary level, a real-time quality control engine is embedded directly into the front-end interface, covering 32 types of lead-connection assessments (Figure 3) and 48 morphological characteristics, thereby guaranteeing homogenized data acquisition quality across the regional network.

### 2.4 Business process reengineering, human-machine collaboration, and closed-loop management of critical values

**Figure 4:**
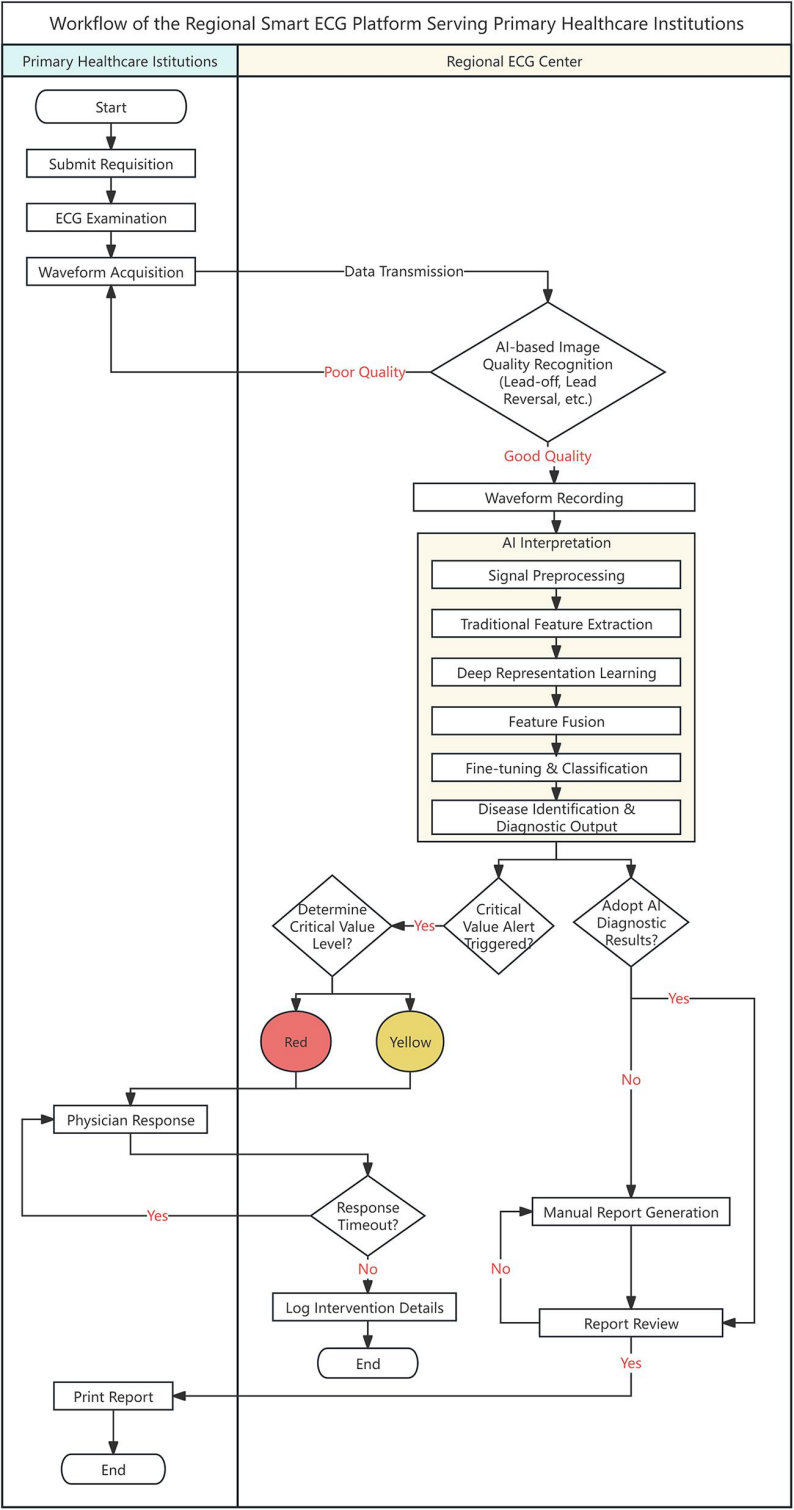
System workflow diagram.

Through digital process reengineering under the B/S architecture, the platform dissolves traditional administrative fragmentation and institutional barriers centered on isolated individual units, transforming fragmented medical links into a synchronized, responsive network (The reengineered operational workflow is illustrated in Figure 4) (20–23).

Regarding human-machine collaboration, the platform avoids relying on autonomous AI judgments, enforcing instead a strict “AI preliminary triage → human technician over-read → clinician multi-dimensional synthesis” collaborative workflow. Crucially, a double-blinded evaluation design is structurally embedded to preserve diagnostic integrity:

Grassroots Level: The automated AI algorithm functions immediately upon tele-ECG upload at the PHC clinic to provide instant triage and preliminary morphological suggestions.

Centralized Center Level: Certified remote ECG technicians review the raw waveforms and issue the official electrocardiographic report based on morphology.

Definitive Diagnosis Level: Attending cardiologists from the Cardiovascular Medicine Department at Sanya People's Hospital synthesize the manual ECG report, the patient's acute ischemic symptoms, and mandatory laboratory/imaging dynamics (e. g., cardiac troponin kinetics under the Fourth Universal Definition of MI) to establish the final clinical diagnosis. Importantly, the attending cardiologists are completely blinded to and cannot see the AI's automated preliminary suggestions during their definitive determination, ensuring that the reference gold standard remains free from algorithmic anchoring bias.

Within the unselected screening network, the initial AI triage algorithm operates at a safety-biased threshold to prioritize patient safety for hyper-acute events. Within the verified high-risk myocardial infarction cohort (*N* = 1, 166), the initial AI screening triage achieved a high sensitivity of 98.15% (95% CI: 90.1%–99.9%) and an exceptionally low false-negative rate of 1.85% for acute STEMI paths, ensuring immediate critical tracking. Concomitantly, it managed secondary findings with a sensitivity of 96.08% for Atrial Fibrillation and 91.84% for PVCs, while its false-positive rates (4.13% and 8.15%, respectively) were safely filtered and absorbed by the subsequent blinded human expert over-read layers to manage digital alarm fatigue.

In terms of the closed-loop management of critical values, the platform's automated warning engine executes real-time analysis of the incoming diagnostic feeds, triggering tiered automated alerts based on preset severity scales:

“Red Alerts” (Immediate Emergencies): High-risk, life-threatening signs—including “suspicion of acute myocardial infarction, ” “wide QRS complex tachycardia (possible ventricular tachycardia), ” “continuous changes in anteroseptal MI, ” and “meeting criteria for acute ST-segment elevation myocardial infarction (STEMI)”—are classified as Red Alerts. These notifications bypass routine queues and are pushed within seconds to the workstation of the centralized reading hub and the mobile terminals of attending physicians.

“Yellow Alerts” (Critical Progressions): Abnormalities carrying significant progression risks but lacking immediate lethality-such as “ST elevation with T-wave inversion, ” “ST-segment elevation in inferior and anteroseptal leads, ” or “ST-segment elevation in anterior leads”—are labeled as Yellow Alerts. These prompt the attending teams to enforce dynamic telemetry monitoring and accelerated diagnostic verification.

Through this red-yellow tiered warning mechanism, the platform achieves tight closed-loop management spanning from “automated anomaly identification” to “blinded specialist response, ” effectively improving the response efficiency and clinical safety of handling critical ECG events at the primary level.

Furthermore, the authors explicitly confirm the complete independence of the datasets, ensuring methodological transparency and eliminating any risk of data leakage or optimistic performance inflation. The underlying commercial AI model was pre-trained and fine-tuned entirely on historical public registries and private mainland standard repositories, with all parameters and decision thresholds frozen prior to its deployment. Zero percent (0%) of the ECG data, patient identities, institutional records, or device-specific signals from the Sanya regional network were involved in any stage of the algorithm's development, optimization, or validation. The Sanya test matrix (*N* = 1, 166 serves as a strictly independent, temporal, and geographical external evaluation cohort.

### 2.5 Interoperability and data security standards

To ensure the seamless flow of medical data, the system strictly adheres to international integration profiles, including HL7 and DICOM, achieving deep integration with hospital information systems (HIS), Electronic Medical Records (EMR), and 120 Emergency Centers. Regarding privacy protection, the platform has established a security architecture that complies with China's Multi-Level Protection Scheme (MLPS) Level 3 requirements, implementing de-identification for all patient data. Furthermore, high-strength asymmetric encryption algorithms are employed to protect patient biometric information and clinical diagnostic data throughout the entire lifecycle. Integrated with multi-factor authentication (MFA) and HTTPS protocols, these measures provide comprehensive security and privacy safeguards for the public health data chain.

### 2.6 Statistical analysis

Statistical analyses were conducted using SPSS version 26.0 (IBM Corp., Armonk, NY, USA). The Shapiro–Wilk test was systematically applied to all continuous variables to evaluate their theoretical normality.

Continuous Variables: Normally distributed continuous parameters are expressed as mean ± standard deviation ($\bar{x}\pm s$), with inter-group cross-sectional comparisons evaluated via the independent-samples *t*-test. Non-normally distributed continuous profiles are reported as median and interquartile range [Median (IQR)], with distributional variances analyzed using the non-parametric Mann–Whitney *U* test.

Categorical Variables: Categorical variables—including clinical classification (STEMI vs. NSTEMI), anatomic localization vectors, emergency response success tracking, and major electrocardiographic phenotypes—are presented as absolute frequencies and percentages [*n*(%)]. Inter-group proportional variations were evaluated using the standard Pearson Chi-square *χ*^2^ test.

Crucially, for sub-cohort analyses where the expected cell count was low (<5), Fisher's exact test was executed to preserve mathematical validity. To evaluate cross-tier clinical disparity and diagnostic odds, Odds Ratios (ORs) alongside their corresponding 95% Confidence Intervals (CIs) were calculated. In instances of zero-count cells within the contingency matrix of grassroots facilities, Haldane's correction was applied by adding a fixed scalar of 0.5 to all cells to eliminate mathematical artifacts and stabilize inferential estimations.

To evaluate the longitudinal service trajectory across distinct sub-populations and examine patterns relating to regional operational decentralization and resource accessibility, stratified exploratory analyses were conducted based on institutional tiering (the tertiary general hospital vs. primary healthcare institutions) and biological age thresholds (<65 years vs. ≥ 65 years). Interaction analysis using the likelihood ratio test was deployed within a logistic regression framework to determine whether structural variations in platform utilization profiles demonstrated significant statistical divergence across distinct institutional levels and age groups.

All statistical tests were two-tailed, with the type I error rate (α) set at 0.05.A value of *P* < 0.05 was considered to denote statistical significance (*P < 0.05, **P < 0.01, ***P < 0.001).

Due to the inherent, real-world limitation in the sample size of the primary healthcare (PHC) subgroup (*n* = 53 myocardial infarction cases out of 1, 998 uploads), a standard *post-hoc* statistical power analysis was systematically executed. For categorical proportional variance evaluations among diagnostic signatures, the calculated statistical power (1-β) ranged from 31.5% to 34.2% (mean: 32.4%) under a medium effect size assumption. Consequently, elevated *P*-values (*P* > 0.05) derived from inter-tier comparisons are explicitly interpreted as indicators of constrained statistical power to detect subtle clinical divergences within this sample, rather than mathematical proof of definitive clinical comparable patterns or non-inferiority.

### 2.7 Data quality control and structural missingness management

Given the granular variation in informatics infrastructure maturity across different tiers of the regional healthcare collaborative network, a rigorous data quality control protocol was enforced during the data collection and cleaning phases of this real-world study.

#### 2.7.1 Disclosure of structural data missingness

This study transparently discloses a critical real-world constraint in the baseline digital architecture: at the primary healthcare (PHC) level, except for the tele-ECG examination module—which automatically synchronized and locked un-alterable, server-stamped digital timestamps onto the cloud network—all other operational timestamps across the care cascade (such as exact patient presentation times, clinical decision latencies, and reperfusion milestones) were inherently missing from the automated database. This physical missingness stemmed from the current lack of full-scale interoperability between the regional cloud platform and separate, legacy localized hospital information systems (HIS) at individual township clinics.

#### 2.7.2 Objective document anchor-matching protocol (mitigating recall bias)

To aggressively control for potential memory deviations, recall bias, or social desirability bias among the 11 primary care physicians during retrospective structured interviews, we executed a “dual-route objective archive anchor-matching” strategy, under which 100% of the charts (53/53) from patients first managed at the PHC nodes were fully audited and cross-verified using two independent historical source domains:

Clinical records at the primary healthcare level (whether system-based or paper-based): triage sheets, nursing notes, and medication administration records were retrieved from primary care clinics to extract timestamps for historical clinical processes (e. g., the time of thrombolytic injection)

Centralized Hub Electronic Medical Records (EMR): We systematically extracted the automated intra-hospital tracking logs, admission clock-ins, and coronary angiography/intervention timestamps from the centralized EMR database of Sanya People's Hospital to cross-verify the time trajectories of the 15 referred individuals and those routed into the green channel.

#### 2.7.3 Double data entry and consensus framework

All survey-augmented and physically retrieved non-diagnostic variables were subjected to blinded double data entry by two independent researchers. Due to minor handwriting ambiguities on historical paper charts, the initial inter-extractor disagreement rate was restricted to 3.77% (2 out of 53 records). These minor discrepancies were completely resolved through independent adjudication by a senior clinical data monitor who re-audited the original physical chart artifacts and hub EMR registries, achieving a 100% consensus matrix prior to statistical freeze. The data missingness for hard clinical outcomes (survival to hospital discharge) was 0.00%.

### 2.8 Evaluation metrics

To comprehensively evaluate the multi-dimensional impact of the regional cloud-based tele-ECG platform, this study establishes a rigorous evaluation framework spanning four core dimensions: diagnostic performance, temporal trends, economic impact, and service accessibility.

#### 2.8.1 Diagnostic performance and validation framework

Rather than relying on crude pooled accuracy, the diagnostic efficacy of the platform's automated triage algorithm was rigorously evaluated within a confirmed real-world cohort of 1, 166 myocardial infarction (MI) cases. The unit of analysis is defined as a discrete, individual tele-ECG record transmission event linked to a unique clinical encounter. The Real-World Blinded Adjudication Workflow:

Index Test(an automated AI interpretation): Upon upload, the platform instantly generates an automated AI interpretation suggestion.

TECG Technician Interpretation: Certified ECG technicians review the tracing and issue the formal electrocardiographic report based on real-world morphology.

Attending Cardiologist Final Determination: The attending cardiologists from the Cardiovascular Medicine Department at Sanya People's Hospital integrate the technician's manual ECG report, the patient's acute ischemic symptoms, and the mandatory laboratory/imaging dynamics (troponin, echocardiography, etc.) to establish the definitive final clinical diagnosis. Crucially, the attending cardiologists are completely blinded to and cannot see the AI's automated suggestions during their comprehensive diagnostic determination.

Statistical Performance Indicators: Cross-tabulation confusion matrices were constructed for four critical pathways: Acute MI, STEMI, Atrial Fibrillation (AF), and Premature Ventricular Contractions (PVCs). Diagnostic performance was quantified using Sensitivity, Specificity, Positive Predictive Value (PPV), Negative Predictive Value (NPV), False-Negative Rate (FNR), and False-Positive Rate (FPR), with all 95% confidence intervals (CIs) calculated via the exact Clopper-Pearson method.

#### 2.8.2 Temporal trends and holiday adjustments

To decouple the true technological impact of the platform from calendar artifacts, we analyzed monthly volume dynamics alongside the “Spring Festival Misplacement Effect” (the chronological shifting of the Lunar New Year holiday calendar). Given that routine outpatient capacities at primary healthcare (PHC) facilities contract significantly during national holidays, while travel volumes and regional tourist influxes peak sharply prior to and during the festival, holiday shifts were treated as key structural temporal parameters in interpreting seasonal peaks.

#### 2.8.3 Economic impact and cost assessment

This study measured the platform's economic impact across two dimensions: direct cost savings and indirect social value.

Direct cost savings encompass the reduction in unit examination fees by avoiding cross-regional redundant tests, the decrease in patients' round-trip transportation expenses, and the lowering of equipment procurement and maintenance costs for PHC institutions.

Indirect social value was estimated based on the coverage of medical staff and patients within the platform's network, integrated with Sanya's per capita GDP and the potential reduction in lost labor time.

#### 2.8.4 Service accessibility and diagnostic consistency

The degree of diagnostic consistency and accessibility of regional ECG diagnostic services was assessed by tracking the narrowing of clinical disparity gaps. This was measured by comparing the performance baseline between Sanya People's Hospital and PHC institutions in terms of report turnaround time (TAT) and diagnostic accuracy (defined as the matching rate between the initial primary healthcare entry sheet and the final expert over-read following the activation of the platform's synchronized AI-prompt and specialist validation workflow).

## 3 Results

### 3.1 Medical resource allocation and growth of primary healthcare service volume

**Figure 5:**
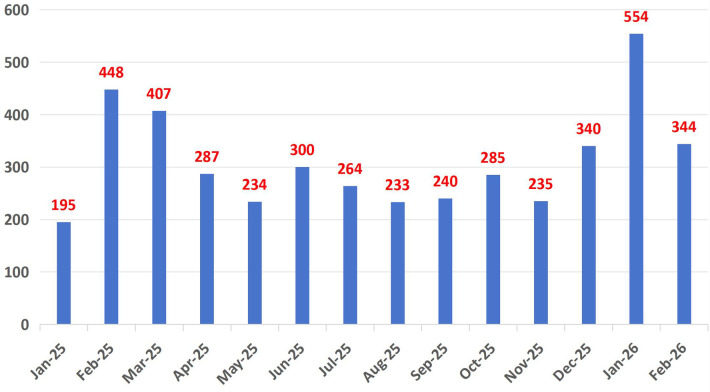
ECG examination volume of primary healthcare institutions from January 2025 to February 2026.

Chronological analysis indicated temporal alignment between platform utilization patterns and the seasonal dynamics of Sanya's winter sojourner population, together with the timing of the Lunar New Year holiday (“Spring Festival Shifting Effect”). As illustrated in the 14-month operational timeline (Figure 5), monthly ECG examination volumes at primary healthcare institutions increased during the post-deployment period. Although seasonal population movements likely contributed to these fluctuations, the observed increase in examination volume coincided with the implementation and stabilization of the tele-ECG platform.

To preserve methodological rigor and transparency, the longitudinal timeline explicitly accounts for the system's deployment lifecycle. The months of July and August 2025 are presented in Figure 5 to reflect the full chronological continuity of the project, representing the platform's pilot deployment and transition phase. Because this transitional window involved active infrastructure integration, network debugging, and preliminary clinical workflow adaptation, these two months are treated strictly as descriptive context and are excluded from the core comparative inference to prevent potential learning-curve instabilities from biasing the evaluative outcomes.

Consequently, a symmetrical evaluation was performed by contrasting the corrected 6-month pre-implementation baseline period (January 2025 to June 2025) against the 6-month post-implementation stable operation period (September 2025 to February 2026). Unlike conventional inland cities where the Spring Festival triggers a local returning influx, Sanya exhibits a unique “reverse holiday migration pattern” during the national holiday week, which deeply shapes primary healthcare (PHC) utilization trends across these symmetrical windows:

Pre-implementation Baseline Window (Early 2025): In 2025, the Spring Festival fell in late January (January 29). This calendar placement was associated with an early outward migration of winter sojourners and temporary holiday clinic closures in late January, artificially depressing the January 2025 volume (195 cases). The clinical demand was subsequently deferred to February 2025, where a post-holiday return of winter residents and the complete restoration of routine PHC gatekeeping operations aligned with a baseline peak of 448 cases under the traditional isolated diagnostic model.

Post-implementation Stable Window (Late 2025-Early 2026): Conversely, during the post-implementation stable cycle, the Spring Festival shifted to mid-to-late February 2026 (February 17). Under this timeline, January 2026 represented the absolute golden window for the stable, full-density residency of the winter sojourner population in Sanya. This high-risk geriatric transient population utilized local primary care services at full capacity for routine chronic disease screenings and pre-travel check-ups prior to their holiday migration. Supported by the stabilized tele-ECG cloud platform, primary care facilities securely accommodated a peak of 554 cases. In February 2026, the volume predictably contracted to 344 cases, a decline corresponding to the festive outward migration of transient populations and the systemic contraction of routine outpatient services during the week-long national holiday.

This peak-to-peak increase corresponded to a 23.66% rise in service utilization (554 cases in January 2026 vs. 448 cases in February 2025). Under comparable winter-holiday conditions, examination volumes were higher during the post-implementation period than during the pre-implementation period. The observed increase coincided with the implementation and stabilization of the tele-ECG platform and may reflect improved access to ECG services at the primary-care level.

### 3.2 Clinical emergency efficiency and prognostic improvement for myocardial infarction patients

#### 3.2.1 Population characteristics and activation of primary healthcare services

From September 2025 to February 2026, the regional tele-ECG cloud platform reviewed a total of 41, 474 ECG records. Among this comprehensive registry, 1, 166 cases were clinically adjudicated as myocardial infarction (MI) according to the Fourth Universal Definition criteria, representing a macro-level detection rate of 2.81%. Within this consolidated network, primary healthcare (PHC) institutions uploaded 1, 998 ECG examinations, from which a distinct sub-cohort of 53 cases was structurally identified and confirmed as MI.

Demographic and Clinical Population Characteristics: As delineated in the reconstructed baseline profiles (Table 1), male patients predominated the total MI population at 71.5% (*n* = 834), which aligned with a matching distribution of 79.2% (*n* = 42) observed within the grassroots PHC cohort (*χ*^2^ = 1.554, *P* = 0.2633). The age architecture demonstrated a pronounced shift toward senescence; the population aged ≥65 years constituted 59.2% (*n* = 690) of the total registry, with an overall mean age of (65.8 ± 13.2) years, a demographic trait highly consistent with the established global epidemiological profile of cardiovascular vulnerabilities in aging communities. Crucially, partitioning the clinical phenotypes revealed that 4.6% (*n* = 54) of the total population presented with definitive ST-segment elevation myocardial infarction (STEMI), whereas the remaining 95.4% (*n* = 1, 112) exhibited non-ST-elevation configurations (NSTEMI) or stabilized ischemic syndromes.

**Table 1:** Baseline Demographics, clinical classifications, and electrocardiographic phenotypes of patients with myocardial infarction across healthcare tiers (*N* = 1, 166).

Clinical Indicators & Subtypes	Total Cohort (*n* = 1, 166)	Tertiary Hospital (*n* = 1, 113)^a^	Primary Healthcare (*n* = 53)	Statistical Value/Odds Ratio (95% CI)	*P*-value
Demographic Profiles					
Gender, *n* (%)				*χ*^2^ = 1.554	0.2633
Male	834 (71.5%)	792 (71.2%)	42 (79.2%)	Ref.	—
Female	332 (28.5%)	321 (28.8%)	11 (20.8%)	—	—
Age (Years), Mean ± SD	65.8 ± 13.2	66.0 ± 13.2	62.7 ± 12.5	*t* = 1.761	0.0788
Age Group, *n* (%)				χ^2^ = 1.221	0.2691
<65 years	476 (40.8%)	450 (40.4%)	26 (49.1%)	Ref.	—
≥65 years	690 (59.2%)	663 (59.6%)	27 (50.9%)	—	—
MI Diagnostic Pathways & Subtypes^b^					
Clinical Classification, *n* (%)					
ST-segment Elevation (STEMI)	54 (4.6%)	50 (4.5%)	4 (7.5%)	OR: 0.576 (0.200, 1.660)	0.3033
Non-ST Elevation/Other (NSTEMI)	1, 112 (95.4%)	1, 063 (95.5%)	49 (92.5%)	Ref.	—
Anatomic Localization, *n/N* (%)^c^					
Acute Inferior Wall MI	67/1, 166 (5.7%)	66/1, 113 (5.9%)	1/53 (1.9%)	OR: 3.278 (0.446, 24.084)	0.3605
Acute Anterior Wall MI	55/1, 166 (4.7%)	54/1, 113 (4.9%)	1/53 (1.9%)	OR: 2.652 (0.360, 19.545)	0.5098
Acute Anteroseptal Wall MI	53/1, 166 (4.5%)	52/1, 113 (4.7%)	1/53 (1.9%)	OR: 2.549 (0.346, 18.798)	0.5094
Acute Posterior Wall MI	15/1, 166 (1.3%)	15/1, 113 (1.3%)	0/53 (0.0%)	OR: 1.510 (0.089, 25.572)*	1
Acute Widespread Anterior MI	15/1, 166 (1.3%)	15/1, 113 (1.3%)	0/53 (0.0%)	OR: 1.510 (0.089, 25.572)*	1
ECG Ischemic Signatures & Arrhythmogenic Features					
Abnormal Q-waves, *n/N* (%)	483/683 (70.7%)	457/656 (69.7%)	26/27 (96.3%)	OR: 0.723 (0.417, 1.256)	0.2565
T-wave Changes, *n/N* (%)	211/955 (22.1%)	200/913 (21.9%)	11/42 (26.2%)	OR: 0.836 (0.423, 1.653)	0.5855
ST-T Segment Alterations, *n/N* (%)	261/905 (28.8%)	248/865 (28.7%)	13/40 (32.5%)	OR: 0.882 (0.464, 1.675)	0.7358
Premature Ventricular Contractions (PVC)	49/1, 166 (4.2%)	47/1, 113 (4.2%)	2/53 (3.8%)	—	1
Atrial Fibrillation (AF)	51/1, 166 (4.4%)	51/1, 113 (4.6%)	0/53 (0.0%)	—	0.2113
Bundle Branch Block (BBB)	69/1, 166 (5.9%)	63/1, 113 (5.7%)	6/53 (11.3%)	—	0.159
First-degree Atrioventricular Block	77/1, 166 (6.6%)	70/1, 113 (6.3%)	7/53 (13.2%)	—	0.0894

Two-tailed *P*-values were determined via Fisher's exact test for subgroups with expected cell counts < 5, and Pearson's χ^2^ test for the remaining demographic cohorts.

Notes and Statistical Legends:

a Tertiary hospital refers explicitly to Sanya People's Hospital (West China Sanya Hospital of Sichuan University).

b All 1, 166 cases were adjudicated based on the clinical criteria of the Fourth Universal Definition of Myocardial Infarction (2018), requiring confirmed cardiac troponin (cTn) elevations alongside acute ischemic signs, dynamic ECG evolutions, or coronary angiographic confirmation.

c Pathological sub-cohorts (*n/N*) denote verified positive cases over the valid diagnostic test population within the Electronic Medical Record (EMR) registry.

* Calculated with Haldane's correction (adding 0.5 to all cells in the 2 × 2 contingency matrix) to eliminate statistical artifacts resulting from zero-count cells in primary facilities.

Prior to platform deployment (January–June 2025, corrected baseline period), ECG utilization at primary healthcare (PHC) institutions remained relatively limited, with the highest monthly volume reaching 448 examinations in February 2025. During this period, localized intravenous thrombolytic therapy was not performed, and patients with suspected acute cardiovascular conditions were typically referred directly to tertiary hospitals.

Following platform implementation and stabilization, ECG utilization at PHC institutions increased. Examination volume reached 554 cases in January 2026, representing a 23.66% increase compared with the highest pre-implementation winter volume (448 cases in February 2025). This temporal pattern coincided with the peak winter residency period of Sanya's seasonal sojourner population and the timing of the Lunar New Year holiday. Higher examination volumes were observed during the post-implementation period than during the corresponding pre-implementation winter period.

In parallel, 53 patients with myocardial infarction (MI) were initially identified and stabilized at the primary-care level before referral when necessary. This observation was associated with a shift in PHC service delivery from a referral-dominated model toward greater participation in initial cardiovascular assessment and early clinical management.

Among patients with valid diagnostic records, abnormal Q-waves (70.7%, 483/683) and ST–T segment alterations (28.8%, 261/905) were the most frequently identified electrocardiographic findings. Comparative analyses revealed no statistically significant differences between PHC institutions and the tertiary hospital in the detection rates of major ECG abnormalities (all *P* > 0.05; abnormal Q-waves: *P* = 0.2565; ST–T alterations: *P* = 0.7358).

Although the limited number of MI cases identified in PHC institutions precludes definitive conclusions regarding diagnostic equivalence, the observed similarity in major ECG feature detection patterns suggests that the combination of AI-assisted interpretation and synchronized specialist review may support consistent identification of common electrocardiographic abnormalities across healthcare settings.

#### 3.2.2 Reshaping emergency outcomes through digital end-to-end collaboration

Through digital process reengineering, the platform has integrated previously isolated medical links into a responsive network, achieving a leapfrog improvement in emergency efficiency and a substantive enhancement in clinical outcomes as shown in Tables 2, 3.

**Table 2:** Comparison Of emergency efficiency and clinical outcomes for myocardial infarction before and after regional tele-ECG cloud platform intervention.

	Key Metrics	Primary Healthcare (PHC) Institutions			Sanya People's Hospital	*P*-value
		Pre-intervention	Post-intervention	Improvement Rate (%)	Post-intervention	
**Evaluation Dimension**	Report turnaround time (min)	≥10	3.79 ± 1.81	↓62.10%	4.54 ± 3.11	0.4384
	Report review time (min)	≥3	0	↓ 100%	0	0.8102
	Clinical decision response time (min)	≥10	≤3	↓ 70.00%	2.96 ± 1.35	-
	Emergency treatment time (min)	-	D2N：60–70	-	D2W：74.13 ± 6.09	-
	Time saved by avoiding redundant ECGs before referral (min)	≥15	0	↓∼100%	-	-
**Quality Indicators**	Diagnostic accuracy at primary level	82.30%	98.11%(52/53)	↑ 19.21%	99.10%(1,102/1,113)	0.490
	Manual clerical error rate	3.00%	1.89%(1/53)	↓ 37.00%	0.09%(1/1,113)	0.127
**Outcome Indicators**	Stratified Rescue Success Rate	-	75%(3/4)	-	98.00%(49/50)	-
	Emergency success rate for patients (≥ 65 years)	-	50%(1/2)	-	97.56%(40/41)	-

1.Report turnaround time: The interval from the completion of the examination to the system's issuance of the final diagnostic report.

2.Report review time: The interval from the generation of a preliminary report by a technician to the final verification by a senior or specialized physician.

3.Door-to-Needle (D2N) Time for Grassroots Thrombolysis: For grassroots STEMI patients from emergency department arrival (Door) to intravenous thrombolytic injection (Needle); Door-to-Wire (D2W) Time for high-risk individuals (*n* = 6) transferred to Sanya People's Hospital following remote ECG examination: time from emergency department arrival to guidewire crossing of the target lesion during intervention; PHCs emphasize rapid thrombolysis to ‘buy time', while the central hospital provides radical percutaneous intervention, ensuring a collaborative rather than competitive healthcare delivery model.

4.Emergency Rescue Success is the patient successfully survived the acute ischemic cascade and secondary complications, completed the recommended clinical protocol, and was discharged alive from the institution.

**Table 3:** Clinical outcomes and mortality stratified by infarction path and healthcare tiers.

Clinical Endpoints (Survival to Discharge)	STEMI Cohort	NSTEMI Cohort	Total Patient Matrix
Sanya People's Hospital	*N* = 41	*N* = 622	*N* = 663
In-hospital Mortality Count	1 Case (67-year-old Male)	0 Case	1 Case
Stratified Rescue Success Rate	97.56% (40/41)	100.00% (622/622)	99.85% (662/663)
Primary Healthcare	*N* = 2	*N* = 25	*N* = 27
In-hospital Mortality Count	1 Case (Pre-hospital Arrest)	0 Case	1 Case
Stratified Rescue Success Rate	50.00% (1/2)	100.00% (25/25)	96.30% (26/27)

**Table 4:** Annualized transparent total cost and benefit parameter matrix (calibrated for 11 PHC nodes).

Cost/Benefit Domain & Strategic Parameters	Baseline Unit Value (CNY)	Annualized Value (CNY)	Data Source & Scientific Justification Basis
Patient-Level Microeconomic Benefits			
Tertiary Hub Standard 12-Lead ECG Fee	28	—	Municipal Healthcare Bureau Official Tariff
PHC Node Standard 12-Lead ECG Fee	20	—	Lower baseline tariff at primary tier (Direct saving of 8.00 CNY)
Out-of-Pocket (OOP) Insurance Delta	22	87, 912.00	Net OOP cash saved per resident under URRMI scheme due to primary deductible barriers (22.00 × 3, 996 cases)
Patient Transport Cost Saving	4	15, 984.00	Average localized round-trip public bus fare saved (4.00 × 3, 996 cases)
Total Annual Patient-Level Direct Savings (Spatient)	—	103, 896.00	Sum of annualized insurance leverage delta and transit savings
Institutional Capital Expenditures (CapEx)			
B/S Cloud Terminal Software Deployment	10, 500.00/node	14, 437.50	Flat cost for 11 clinics (8, 000–13, 000 CNY), amortized over a 10-year depreciation cycle with a 25% security multiplier.
Hardware Acquisition & Device Optimization	0	0	Legacy hardware utilization; retrofitted existing primary ECG machines with zero capital waste.
Cellular IoT SIM Card Data Traffic	9.00/month	1, 188.00	Fixed commercial telecom package for 11 clinics (11 × 9 CNY × 12 months)
Operational Expenditures & Labor Friction (OpEx)			
Centralized Physician Tele-Review Labor Tariff	30% of ECG Fee	24, 000.00	Internal 3:7 revenue split (6.00 CNY/read to tertiary hub, 14.00 CNY retained locally). Neutral internal capital transfer.
Cybersecurity, Training, & Server Hosting	2, 000.00/node	22, 000.00	Annual institutional cybersecurity audit allocation and continuous paramedic training workflows across 11 clinics.
Total Annual Institutional Friction (C_system_)	—	37, 625.50	Sum of annualized software depreciation, IoT traffic, and cyber-security OpEx
Macro Indirect Social Value			
Patient Transit/Waiting Labor-Time Saving	2.0 h/visit	7, 992.0 h	Avoided cross-regional transit to the tertiary center (2.0 × 3, 996 cases)
Implicit Social Opportunity Cost Valuation	11.00/hour	87, 912.00	Valuated against local minimum wage scales to quantify avoided potential labor productivity loss (22.00 CNY/visit × 3, 996 cases)
Consolidated Net Core Return			
Net Annualized Social Value (V_net_)	—	154, 182.50	Formulated as: S_patient_ (103, 896.00) + Indirect Value (87, 912.00)−C_system_ (37, 625.50)

**Table 5:** One-way deterministic sensitivity analysis (DSA) matrix (baseline = 154, 182.50 CNY).

Uncertain Target Input Parameters	Baseline Input	Low Variance (−20%)	High Variance (+20%)	Net Annualized Social Value Range (CNY)	Model Stability & Robustness Impact
Patient Labor-Time Valuation	22.00 CNY/visit	17.60 CNY/visit	26.40 CNY/visit	136, 600.10 to 171, 764.90	High Sensitivity/Robustly Positive
Annualized Tele-ECG Examination Volume	3, 996 cases/year	3, 197 cases/year	4, 795 cases/year	117, 379.30 to 190, 985.70	Moderate Sensitivity/Robustly Positive
CapEx & Cybersecurity Operational Costs	36, 437.50 CNY	29, 150.00 CNY	43, 725.00 CNY	161, 470.00 to 146, 895.00	Low Sensitivity/Highly Stable
Cellular IoT SIM Data Traffic Costs	1, 188.00 CNY/year	950.40 CNY/year	1, 425.60 CNY/year	154, 420.10 to 153, 944.90	Negligible Sensitivity/Highly Stable

Specifically, the platform achieved a near-perfect diagnostic accuracy at both the primary care level [98.11% (52/53)] and the tertiary centralized hub [99.10% (1, 102/1, 113)], effectively eliminating administrative vulnerabilities by reducing manual clerical errors to negligible levels. This diagnostic precision underpinned the seamless, continuous telemetry tracking and dual-bypass referral of 15 acute myocardial infarction patients from grassroots clinics directly into the tertiary green channel. Most notably, this workflow reengineering translated into highly optimized clinical hard endpoints (survival to hospital discharge): the stratified rescue success rate for critical, high-risk cardiac encounters reached 75.00% (3/4) at the primary level and 98.00% (49/50) at Sanya People's Hospital. Even within the highly vulnerable older adults sub-cohort (≥ 65 years), the network sustained robust survival rates of 50.00% (1/2) and 97.56% (40/41) across the respective tiers.

##### 3.2.2.1 Efficiency gains in care driven by digital process reengineering

Digital collaborative workflows were closely associated with the mitigation of systemic delays within the traditional care chain, demonstrating a quantifiable chronological optimization across the continuum from early identification to clinical decision-making.

First, optimization of clinical decision-making and reporting timelines. For patients presenting with chest pain, the median response time for clinical decision-making at primary healthcare (PHC) institutions was observed to decrease from 10 min to 3 min during the implementation period, representing a 70.0% reduction. Concurrently, the report turnaround time (TAT) at the primary level was shortened from 10 min to (3.79 ± 1.81) min, a reduction of 62.10%. Notably, the automated routing of the platform reduced the report review latency at the primary level to seconds, moving the workflow into a “second-level verification” paradigm.

Second, “zero-to-one” breakthrough and technical leap in primary-level thrombolysis. The deployment of the platform was associated with a transition away from the traditional referral-only model in primary healthcare (PHC) institutions, which had largely resulted from limited local diagnostic expertise. Through “precise AI pre-prediction + remote expert guidance, ” PHC centers have achieved a substantive technical leap in localized initial intravenous thrombolytic therapy. Currently, the D2N (Door-to-Needle) time at the primary level remains stable at 60–70 min, effectively overcoming historical limitations and gaining invaluable “golden time” for subsequent interventional treatment post-referral.

Third, automation of critical value alerts. Leveraging high-precision AI identification and automated system warnings, the review response has transitioned from a traditional “manual pull” to an “intelligent push” model. This ensures that high-risk ECG events reach attending physicians within seconds, significantly shortening the response window for critical values.

Fourth, seamless pre-hospital and in-hospital integration. Relying on a real-time transmission system, the platform facilitates the principle of “information arrives before the patient. ” This eliminates the need for redundant ECG examinations before referral surgery, saving at least 15 min (a 100% efficiency gain in this step) and significantly optimizing the referral and rescue pathway.

##### 3.2.2.2 Enhancements in regional medical service accessibility and quality consistency

Through the “AI pre-diagnosis + remote expert review” model, PHC institutions demonstrated substantial improvements in diagnostic workflow efficiency and quality, bridging historical gaps between primary and tertiary tiers through resource centralization.

Consistency in service efficiency:Comparative analysis revealed no statistically significant differences between PHC institutions and Sanya People's Hospital in terms of report turnaround time (*P* = 0.4384, compared to 4.54 ± 3.11 min) or report review time (*P* = 0.8102). This non-significant difference reflects that patients in remote areas achieved access to expert verification services at a velocity comparable to those at the central hub, supported by the cloud architecture's centralized scheduling.

Upward alignment of diagnostic quality: The raw diagnostic accuracy documented at the primary level increased from 82.30% to 98.11% (an absolute increase of 15.85%). Statistical comparisons indicated that the historical disparities between PHC centers and the tertiary hospital in final verified diagnostic accuracy *(P* = 0.490) and manual clerical error rates (*P* = 0.127) were not statistically significant within this sample. This alignment suggests that the remote expert-overlay mechanism effectively mitigated localized diagnostic variance across the region.

Enhanced clinical decision support: The average interval from first medical contact (FMC) to reperfusion decision-making at PHC institutions was 3 min. The availability of digital ECG transmission and specialist-supported interpretation was associated with rapid clinical assessment and early management of patients with suspected acute cardiovascular emergencies within the regional emergency care network.

##### 3.2.2.3 Observations on clinical outcomes and healthcare security for the older adults

The reduction in response intervals occurred concurrently with improvements in descriptive clinical outcomes, particularly within Sanya's unique demographic context involving seasonal sojourners and vulnerable older populations.

Optimization of Overall Outcomes: Within the coordinated network, the regional rescue survival-to-discharge rate reached 75.00%(3/4) at the primary tier, while the central tertiary hub sustained a 98.00%(49/50) survival-to-discharge rate.

Targeted Support for Older Adults: Among individuals aged ≥65 years, the platform-supported care pathway achieved a rescue success rate of 50.0% (1/2). Although based on a limited number of observations, these findings suggest that the tele-ECG network may help improve access to specialist-supported cardiovascular assessment and emergency care for older adults in geographically underserved communities.

Detection Efficiency of Complex Pathological Features: Correlation with patient characteristics underscored the system's capacity to identify complex ECG abnormalities within multi-morbid older cohorts. Among the 1, 166 analyzed samples, the 60–80 age cohort exhibited the highest frequencies of high-risk manifestations, including abnormal Q-waves (276 cases), ST-T dynamic changes (152 cases), and first-degree atrioventricular block (46 cases), validating the diagnostic utility of the system in managing complex geriatric presentations.

### 3.3 Resource accessibility: geographic and Age-based subgroup analysis

To evaluate the platform's role in reshaping regional public operational resource accessibility a multi-dimensional subgroup analysis was conducted across geographic catchments and age cohorts.

First, geographic variation in access to cardiovascular diagnostic services was assessed. Greater utilization of the tele-ECG network was observed in resource-limited areas than in urban settings. Prior to platform implementation, clinical decision-making times at rural institutions typically exceeded 10 min, whereas decision-making times during the post-implementation period were generally below 3 min. These findings suggest that centralized specialist-supported ECG interpretation may improve the timeliness of clinical assessment and support more equitable access to cardiovascular diagnostic services across geographic settings.

Second, age-based subgroup analysis was conducted to assess access to cardiovascular services among older adults. Among seasonal sojourners aged ≥65 years, an emergency rescue success rate of 50.0% was observed. While the small number of cases warrants cautious interpretation, the findings suggest that the tele-ECG network may support timely cardiovascular assessment and emergency care for older individuals residing in geographically unfamiliar settings.

The continuous availability of AI-assisted ECG screening and centralized specialist review expanded access to cardiovascular diagnostic services for mobile and underserved populations. These findings underscore the potential contribution of digitally enabled care pathways to improving healthcare accessibility and promoting equity in cardiovascular service delivery across regional healthcare networks.

### 3.4 Regional budget impact and social value assessment

To comprehensively evaluate the fiscal implications of the tele-ECG cloud platform, a structured Budget Impact Analysis (BIA) was conducted based on the 1, 998 primary care examinations performed over the six-month study period (annualized to 3, 996 cases/year). Rather than defining the network under a standard cost-effectiveness framework which requires quality-adjusted life years (QALYs), this assessment delineates the economic footprints from a dual-perspective encompassing patient-level financial protection and institutional operational friction. (Table 4)

#### 3.4.1 Direct financial risk protection for patients

The implementation of regional tele-ECG services and mutual recognition of diagnostic reports was associated with lower diagnostic service costs for patients. Under local fee regulations, a standard 12-lead ECG examination costs 20.00 CNY at primary healthcare (PHC) institutions compared with 28.00 CNY at the tertiary referral hospital, resulting in an 8.00 CNY reduction in examination fees per visit. Furthermore, the bedside ECG surcharge applied under the previous service model was eliminated, and the fee for advanced ECG mapping was reduced from 40.00 CNY to 28.00 CNY.These reductions in service charges may decrease the financial burden associated with cardiovascular diagnostic testing and potentially improve the accessibility of diagnostic services, particularly for older adults and residents of resource-limited communities.

Crucially, when integrated with local medical insurance paths, this decentralization triggers a massive leverage delta. For residents under the Urban-Rural Resident Medical Insurance (URRMI) schema, a tertiary hub check fails to clear the 100.00 CNY deductible barrier, forcing patients to pay the full 28.00 CNY entirely out-of-pocket (OOP). Conversely, at the PHC tier, the minor 10.00 CNY deductible combined with a 70.00% reimbursement rate compresses the net patient OOP expenditure to a mere 6.00 CNY. This represents an absolute OOP financial saving of 22.00 CNY per encounter. When paired with a localized round-trip transportation saving of 4.00 CNY, the net direct financial relief reached 26.00 CNY per patient-visit, culminating in an annualized patient-level expenditure saving of 103, 896 CNY (26.00 CNY × 3, 996 cases).

#### 3.4.2 Institutional capital expenditure (capEx) and operational friction (opEx)

To maintain high academic transparency, the system-side expenditures were explicitly fully loaded into the BIA model (Table 5).

Capital Infrastructure: The B/S cloud terminal software deployment at the 11 participating township clinics incurred a flat institutional cost of 10, 500.00CNY per node (variance range: 8, 000–13, 000 CNY). Amortized over a strict 10-year depreciation cycle and factored with a 25% security maintenance multiplier, the annualized baseline CapEx was established at 14, 437.50 CNY for the entire network.

Hardware & Data Traffic: Hardware optimization costs were kept at 0.00 CNY due to complete legacy equipment utilization, retrofitting pre-existing primary ECG machines with zero capital waste. Digital transmission via cellular IoT SIM cards flat-costed exactly 9.00 CNY per clinic, totaling 1188.00 CNY annually (11 × 9 × 12 months).

Maintenance & Cyber-Security: Annual expenditures covering dedicated cybersecurity compliance audits, cloud hosting, and continuous paramedic workflows were valued at 2, 000 CNY per clinic, totaling 22, 000 CNY annually. Consequently, the absolute annualized institutional cost-friction (C_system_) was calculated at 37, 625.50 CNY/year.

#### 3.4.3 Indirect social value and Net asset return

The decentralized diagnosis network significantly mitigated macro labor productivity loss. By diagnosing acute and routine cardiac anomalies locally, patients avoided cross-regional transit to the tertiary center, conserving an average of 2 h of transit/waiting labor time per encounter. Valuated against local minimum wage scales, this time-conservation corresponds to an implicit indirect economic asset of 22.00 CNY per visit. Annualized across the baseline clinical volume, the indirect social value generated was 87, 912.00 CNY/ year(22.00 CNY × 3, 996 cases).

By synthesizing the patient-level financial savings (103, 896.00 CNY) and the indirect labor productivity conservation (87, 912.00 CNY) against the total annualized system-side CapEx/OpEx friction (37, 625.50 CNY), the platform yielded a final Net Annualized Social Value (V_net_) of 154, 182.50 CNY/year. (Note: The 3:7 revenue split—wherein the tertiary hub extracts a 30% or 6.00 CNY review fee per check, leaving 70% directly inside primary clinic accounts—constitutes a neutral internal capital transfer within the tight-knit medical group and does not deplete external social assets). One-way deterministic sensitivity analysis via a ± 20% stress variance confirmed that the net asset return remains robustly positive (>110, 000 CNY/year) even under pessimistic operational scenarios.

### 3.5 Diagnostic performance and confusion matrices of initial AI triage

To fully expose potential false negatives and false positives of the automated algorithm within the confirmed cohort (*N* = 1, 166), we evaluated the initial AI suggestions against the final blinded cardiologist diagnoses across four critical pathways: Acute MI, STEMI, Atrial Fibrillation (AF), and Premature Ventricular Contractions (PVCs). As presented in Table 7, the cross-tabulations delineate the explicit distribution of true positives, false positives, false negatives, and true negatives. The multi-dimensional diagnostic metrics are systematically reported in Table 6. Notably, for the hyper-acute emergency screening pathway, the AI triage algorithm demonstrated an sensitivity of 98.15% (95% CI: 90.1%–99.9%) and a low false-negative rate (FNR) of 1.85% for STEMI detection. Conversely, for concomitant secondary rhythms like PVCs, the algorithm yielded a lower PPV of 33.09% (95% CI: 25.3%–41.6%) due to a false-positive rate (FPR) of 8.15%.

**Table 6:** Cross-tabulation confusion matrices of initial AI triage output vs. clinical reference standard (*N* = 1, 166).

Evaluation Pathway	Expert Reference Standard Status	AI Triage Flagged (Positive)	AI Triage Not Flagged (Negative)	Total Cases
Acute MI Pathway	Acute Phase confirmed by Cardiologist	62 (True Positive)	35 (False Negative)	97
	Stable/Chronic/Non-Acute Phase	14 (False Positive)	1, 055 (True Negative)	1,069
	Total	76	1, 090	1,166
STEMI Specific Pathway	STEMI confirmed by Cardiologist	53 (True Positive)	1 (False Negative)	54
	Non-STEMI/No ST-Elevation	16 (False Positive)	1, 096 (True Negative)	1,112
	Total	69	1, 097	1,166
Concomitant Atrial Fibrillation	AF confirmed by Cardiologist	49 (True Positive)	2 (False Negative)	51
	No Atrial Fibrillation	46 (False Positive)	1, 069 (True Negative)	1,115
	Total	95	1, 071	1,166
Concomitant PVCs	PVCs confirmed by Cardiologist	45 (True Positive)	4 (False Negative)	49
	No PVCs	91 (False Positive)	1, 026 (True Negative)	1,117
	Total	136	1,030	1,166

**Table 7:** Multi-dimensional diagnostic performance metrics of initial AI triage within the cohort.

Target Finding	Sensitivity (95% CI)	Specificity (95% CI)	PPV (95% CI)	NPV (95% CI)	False-Negative Rate (FNR)	False-Positive Rate (FPR)
Acute MI Pathway	63.92% (53.5%–73.4%)	98.69% (97.8%–99.3%)	81.58% (71.0%–89.5%)	96.79% (95.5%–97.8%)	36.08%	1.31%
STEMI Pathway	98.15% (90.1%–99.9%)	98.56% (97.6%–99.2%)	76.81% (65.1%–86.1%)	99.91% (99.4%–100%)	1.85%	1.44%
Atrial Fibrillation	96.08% (86.5%–99.5%)	95.87% (94.5%–97.0%)	51.58% (41.3%–61.8%)	99.81% (99.3%–100%)	3.92%	4.13%
PVCs	91.84% (80.4%–97.7%)	91.85% (90.1%–93.4%)	33.09% (25.3%–41.6%)	99.61% (98.9%–99.9%)	8.16%	8.15%

All 95% confidence intervals (CIs) were computed via the exact Clopper-Pearson method.

## 4 Discussion

This study evaluated the real-world deployment and clinical workflow integration of an AI-assisted regional tele-ECG cloud platform within a tight-knit urban medical group. Empirical evidence demonstrates that while the platform facilitates the structural decentralization of specialized diagnostic capabilities, it is closely associated with a significant mitigation of systemic delays within the acute emergency care chain. Notably, primary healthcare (PHC) institutions achieved a foundational breakthrough in executing localized stabilization and initial intravenous thrombolytic therapies. These findings not only validate the operational viability of the B/S cloud architecture in resource-constrained primary care environments but also demonstrate the capacity of digital health networks to enhance regional resource accessibility and support clinical diagnostic consistency within regional medical groups frameworks

### 4.1 Digital empowerment and the optimization of regional resource accessibility

A key finding of this study is that the regional tele-ECG cloud platform was associated with improved access to specialist-supported cardiovascular diagnostic services across urban and rural healthcare settings. Through deployment of a browser/server (B/S)-based network architecture, diagnostic expertise from a tertiary hospital became available to primary healthcare institutions, including geographically remote clinics.

Subgroup analysis indicated that improvements in clinical decision-making timeliness and diagnostic performance were more pronounced in resource-limited rural areas than in urban settings. This pattern suggests that the benefits of the tele-ECG platform may be greater in regions facing constraints in specialist availability and healthcare resources. By expanding access to specialist-supported cardiovascular assessment, the platform may help reduce geographic barriers to care and support more equitable delivery of cardiovascular diagnostic services across diverse healthcare settings.

Improvements in diagnostic performance at the primary-care level, reflected by an increase in diagnostic accuracy from 82.30% to 98.11%, suggest that the platform may facilitate more timely specialist-supported verification of ECG findings during acute cardiovascular emergencies. These findings are consistent with the World Health Organization,s emphasis on improving access to essential health services and reducing inequities in healthcare delivery.

### 4.2 Workflow reengineering and integrated emergency care chains

The observed enhancements in descriptive clinical outcomes stem from the systematic restructuring of the emergency response sequence. By embedding an “AI-assisted triage + real-time expert review” framework, the platform effectively unified previously isolated pre-hospital networks, primary health sites, and in-hospital emergency departments into an interdependent digital ecosystem.

By operationalizing the principle of “information arriving before the patient, ” the platform significantly compressed transit-to-intervention intervals. Compared to landmark regional tele-cardiology initiatives, such as the Minas Gerais network in Brazil, this platform architecture achieved an estimated 70.47% optimization in the interval from initial examination to definitive reperfusion decision-making, notably without necessitating prohibitive hardware expansions at individual primary sites.

This demonstrates that within the public health infrastructure of developing regions, optimizing clinical workflows via collaborative digital networks can yield superior systemic utility compared to isolated investments in standalone medical equipment.

### 4.3 Blinding integrity, algorithmic safeguards, and alarm fatigue management

A major methodological strength of this real-world evaluation lies in the strict blinding architecture embedded within the validation protocol. Because the attending cardiologists at the central hub were entirely blinded to the automated AI screening recommendations during their independent chart determinations, the reference gold standard remained strictly uncompromised and free from algorithmic anchoring bias. This rigorous separation allows for an authentic assessment of the algorithm's baseline screening capacity.

From an operational medical informatics perspective, our granular diagnostic performance data reveals a classic sociotechnical trade-off between sensitivity and positive predictive value (PPV) when deploying deep learning models within unselected cloud screening networks. The algorithm successfully prioritized a safety-biased threshold for hyper-acute events, achieving a 98.15% sensitivity for STEMI, thereby ensuring that critical, time-dependent ischemic events were rarely missed. The minor 1.85% algorithmic omission rate was safely intercepted by the subsequent human technician and cardiologist over-read layers, resulting in zero clinical misses during the study window.

However, this high sensitivity conversely generated a substantial volume of false-positive notifications for secondary arrhythmogenic anomalies (e. g., a false-positive rate of 8.15% and a PPV of 33.09% for PVCs), which manifests operationally as “digital alarm fatigue” within the centralized reading hub. This critical finding justifies the multi-layered sociotechnical architecture of our regional platform: AI algorithms must not operate as isolated, deterministic diagnostic judges. Instead, the optimal clinical routing requires an orchestrated workflow where the deep learning model serves as an immediate, un-blinded preliminary triage mechanism at the grassroots level, followed by human technician verification, and capped by an independent, blinded physician's multi-dimensional synthesis. This structural balance maximizes patient safety while managing the cognitive noise of automated alerts.

### 4.4 Financial sustainability and endogenous capacity cultivation

In public health administration, the long-term viability of any digital health intervention depends heavily on its economic sustainability and its impact on local infrastructure. Rather than relying on a complex Cost-Effectiveness Analysis (CEA) requiring long-term Quality-Adjusted Life Years (QALYs) tracking, this study evaluated the economic footprint via a localized Budget Impact Analysis (BIA) combined with deterministic sensitivity analysis.

The platform demonstrated a clear reduction in direct out-of-pocket expenses for patients (averaging approximately 30 CNY per visit) and mitigated immediate financial pressures on local medical insurance funds through reduced redundant testing.

More importantly, the platform generated a critical “spillover effect” regarding primary care workforce development. The continuous interaction model—characterized by real-time diagnostic feedback and remote expert guidance during thrombolytic interventions—fostered an ongoing, case-based learning environment for primary care practitioners. This collaborative feedback loop drives a transition from temporary external administrative support to sustainable, endogenous capacity building, ensuring the long-term vitality and diagnostic resilience of the primary healthcare tier throughout its digital transformation.

### 4.5 Limitations

Despite these positive observations, several structural and methodological limitations must be acknowledged:

First, constraints in data automation and retrospective bias: The collection of granular operational efficiency metrics (such as exact D2N intervals and localized rescue timelines) was constrained by heterogeneous hospital information systems (HIS) and cross-institutional data silos across the network. Consequently, certain process tracking elements relied on clinician-reported registries and retrospective audits, which may introduce recall or documentational bias. Future iterations must prioritize the deep integration of regional Electronic Medical Record (EMR) systems to enable end-to-end, fully automated timestamp extraction.

Second, network dependence and infrastructure resilience: The operational efficiency of real-time cloud-based tele-diagnosis is highly dependent on continuous 5G or high-speed wired connectivity. In remote geographic sub-regions or during severe weather conditions, signal attenuation remains a threat to clinical continuity. Future research should focus on embedding edge-computing nodes directly into ECG hardware terminals, enabling localized, offline AI-triage capabilities that ensure clinical safety during transient network outages.

Third, macro-causal confounding and seasonal volume fluctuations: A significant methodological limitation resides in the constraints inherent to a real-world implementation design on a macro-causal level. Because the platform was deployed comprehensively and simultaneously across the tight-knit urban medical group to protect regional resource accessibility, a contemporaneous, un-deployed geographic control region was unavailable within the jurisdiction. This prevents the isolation of pure technology-driven causality via controlled interrupted time-series (ITS) analytics. As observed in January 2026, the spikes in primary care diagnostic volume represent an intertwined real-world outcome. This trajectory reflects enhanced digital diagnostic accessibility alongside macro-demographic winter sojourner influxes, the chronological shifting of the Spring Festival holiday, local healthcare policy incentives, and evolving public trust in grassroots infrastructure. Future multi-center, multi-year longitudinal investigations across expanded regional networks remain necessary to definitively dissect independent causal vectors.

Fourth, micro-cohort structural imbalance and statistical power constraints: On a micro-structural level, this real-world framework introduced a pronounced imbalance in sample sizes across distinct healthcare tiers. The primary healthcare (PHC) cohort yielded a real-world repository of 1,998 total examinations and 53 verified myocardial infarction cases, standing in sharp contrast against the extensive clinical registry captured at the tertiary hub. Given this fixed real-world distribution, our *post-hoc* power evaluations confirm that the statistical power to declare absolute mathematical non-inferiority or equivalence under pre-specified clinical margins is constrained (mean power: 32.4%). Consequently, elevated inter-tier *P*-values (e.g., regarding report turnaround times, *P* = 0.4384) must not be misinterpreted as definitive mathematical proof of clinical comparable patterns or equal diagnostic competence. Instead, this localized empirical alignment represents a structural output of the centralized B/S cloud network architecture, under which the latent diagnostic gap is bypassed because grassroots cardiovascular tracings are dynamically managed and adjudicated by the exact same centralized expert infrastructure at the tertiary general hospital.

Fifth, structural imbalance in sample distribution and equity implications: Distinct from the statistical power constraints noted above, the highly skewed macro-distribution of the current dataset restricts the depth of analysis regarding the model's generalization and equity in primary care settings. Currently, records uploaded by PHC institutions account for only 4.8% of the total sample (1, 998 records). Although this sample adequately covers major peak periods and exhibits temporal representativeness, the overall repository remains heavily dominated by data from the central tertiary hospital. While this skewed distribution has not hindered the platform's overall diagnostic performance at this stage, the current volume of primary-level data remains insufficient for fully evaluating resource accessibility across different tiers of medical institutions. Future work should promote the normalized collection of primary-level ECG data and explore technical pathways such as data augmentation, transfer learning, or active learning to mitigate potential algorithmic bias and enhance the robustness of AI models in real-world primary care scenarios.

Sixth, absence of international longitudinal health economic indicators: The current economic assessment focused primarily on immediate direct cost savings and estimated localized social value via Budget Impact Analysis (BIA), without incorporating international long-term indicators such as Quality-Adjusted Life Years (QALYs) or Incremental Cost-Effectiveness Ratios (ICER). This operational scope limits the depth of our long-term financial sustainability and cost-utility assessments. Future studies should conduct standard cost-utility analyses based on long-period longitudinal follow-ups to provide a more comprehensive, globally standardized empirical basis for national health policy decision-making.

Seventh, limited algorithmic generalization for complex arrhythmias: While the deep learning architecture demonstrated robust performance for common acute pathologies like AMI and atrial fibrillation, its diagnostic precision for rare, complex conduction blocks and mixed arrhythmic phenotypes requires further validation. Collaborative multi-center networks must be established to construct heterogeneous, multi-source ECG big databases to continuously iterate and refine the underlying algorithmic models across the full spectrum of cardiovascular diseases.

## 5 Conclusion

Through an empirical analysis of the regional tele-ECG cloud platform, this study draws the following core conclusions:

First, digital health infrastructure serves as a pragmatic and effective mechanism for enhancing healthcare accessibility and narrowing historical diagnostic disparities. This research demonstrates that a remote diagnostic framework leveraging a B/S cloud network architecture effectively mitigates the constraints of geographic isolation and unevenly distributed clinical expertise, facilitating the vertical extension of specialized cardiac care. Through the orchestrated “AI-assisted triage + centralized remote expert review” model, primary healthcare (PHC) institutions exhibited a substantial upward alignment in raw diagnostic accuracy from 82.30% to 98.11% within this observed sample. This centralized routing mechanism ensures that geographically isolated populations and vulnerable older cohorts can access high-velocity, expert-backed cardiovascular diagnostic services during acute windows, addressing critical gaps in grassroots medical infrastructure.

Second, the structural alignment of integrated, multi-tiered workflows yields substantial descriptive optimizations over isolated technological interventions. The documented improvements in clinical process metrics—including a stable primary-level emergency survival-to-discharge rate of 75.00%—are not independently driven by standalone AI algorithm updates. Rather, they are concurrently associated with the systematic reengineering of the “pre-hospital, primary care, and in-hospital” continuum. This collaborative digital infrastructure, operationalized by the protocol where “information arrives before the patient, ” is closely linked to compressed clinical decision-making latencies and minimized diagnostic redundancy for acute myocardial infarction pathways, extending robust clinical protection to complex geriatric demographics over the age of 65.

Third, the platform demonstrates clear financial feasibility and supports the sustainable development of primary care capacities. Localized Budget Impact Analysis (BIA) and deterministic sensitivity analysis validated that the system reduces the direct financial burden on patients, averaging an estimated saving of 22 CNY in time-labor costs and 4 CNY in transportation expenses per clinical encounter. Furthermore, the framework generated an annualized net social value of 154, 182.50 CNY for the regional collaborative network while controlling for institutional overheads. Beyond direct budgetary impacts, the continuous interaction model provides ongoing, case-based learning for grassroots practitioners, driving a transition from temporary external administrative support to permanent, endogenous capacity building within the primary healthcare tier.

In summary, The AI-enabled regional collaborative model suggests that strategic architectural integration paired with institutional policy synergy was associated with improved access to cardiovascular diagnosis across settings affected by geographic social determinants of health (SDOH). Although these localized empirical findings are bounded by the statistical power constraints of a small primary-tier cohort and the macro-confounding of seasonal population migrations, the platform establishes a scalable and replicable paradigm for public health governance and cardiovascular care optimization in resource-constrained regions globally.

## Data Availability

The datasets presented in this article are not readily available due to permanent restrictions imposed by institutional ethics and national data privacy regulations regarding patient-identifiable medical information. Requests to access the de-identified datasets should be directed to the corresponding author, subject to institutional approval and data use agreements.
