# Correction: AI-enabled regional tele-ECG cloud platform and improving access to cardiovascular diagnosis: real-world evidence from southern China

**DOI:** 10.3389/fcvm.2026.1927426

**Published:** 2026-07-16

**Authors:** Jia Xu, Min Pan, Lin Chen, Juan Fu, Rui Shi, Jun Xie

**Affiliations:** 1 Department of IT & Data Management of West China Hospital, Sichuan University, Chengdu, China; 2 Laboratory of Medical Artificial Intelligence, West China Hospital, Sichuan University, Chengdu, China; 3 Department of Cardiovascular Medicine, Sanya People’s Hospital, Sanya, China; 4 West China (Sanya) Hospital, Sichuan University, Sanya, China; 5 Department of Cardiocerebral Function, Sanya People’s Hospital, Sanya, China; 6 Information Center, Sanya People’s Hospital, Sanya, China

**Keywords:** artificial intelligence, budget impact analysis, digital health infrastructure, healthcare accessibility, real-world evidence, tele-cardiology, workflow reengineering

Author Rui Shi was erroneously omitted as corresponding author.

The correct corresponding authors are Rui Shi and Jun Xie.

The original version of this article has been updated.

